# Diabetes impairs the angiogenic potential of adipose-derived stem cells by selectively depleting cellular subpopulations

**DOI:** 10.1186/scrt468

**Published:** 2014-06-18

**Authors:** Robert C Rennert, Michael Sorkin, Michael Januszyk, Dominik Duscher, Revanth Kosaraju, Michael T Chung, James Lennon, Anika Radiya-Dixit, Shubha Raghvendra, Zeshaan N Maan, Michael S Hu, Jayakumar Rajadas, Melanie Rodrigues, Geoffrey C Gurtner

**Affiliations:** 1 Hagey Laboratory for Pediatric Regenerative Medicine; Department of Surgery, Stanford University School of Medicine, 257 Campus Drive West, Hagey Building GK-201, Stanford, CA 94305-5148, USA; 2 Biomaterials and Advanced Drug Delivery Center, Stanford University, 1050 Arastradero Road, building A, Room A163 Palo Alto, Stanford, CA 94304, USA

## Abstract

**Introduction:**

Pathophysiologic changes associated with diabetes impair new blood vessel formation and wound healing. Mesenchymal stem cells derived from adipose tissue (ASCs) have been used clinically to promote healing, although it remains unclear whether diabetes impairs their functional and therapeutic capacity.

**Methods:**

In this study, we examined the impact of diabetes on the murine ASC niche as well as on the potential of isolated cells to promote neovascularization *in vitro* and *in vivo*. A novel single-cell analytical approach was used to interrogate ASC heterogeneity and subpopulation dynamics in this pathologic setting.

**Results:**

Our results demonstrate that diabetes alters the ASC niche *in situ* and that diabetic ASCs are compromised in their ability to establish a vascular network both *in vitro* and *in vivo*. Moreover, these diabetic cells were ineffective in promoting soft tissue neovascularization and wound healing. Single-cell transcriptional analysis identified a subpopulation of cells which was diminished in both type 1 and type 2 models of diabetes. These cells were characterized by the high expression of genes known to be important for new blood vessel growth.

**Conclusions:**

Perturbations in specific cellular subpopulations, visible only on a single-cell level, represent a previously unreported mechanism for the dysfunction of diabetic ASCs. These data suggest that the utility of autologous ASCs for cell-based therapies in patients with diabetes may be limited and that interventions to improve cell function before application are warranted.

## Introduction

Diabetes mellitus (DM) is associated with significant impairments in neovascularization and wound healing [1]. Although the exact mechanism underlying this pathology remains unknown, there is evidence for diabetes-associated dysfunction at both the cellular and molecular level. Specifically, diabetes has been linked to impairments in the functionality of diabetic endothelial progenitor cells (EPCs) and resident tissue fibroblast *in vitro*[2,3]. Additionally, reduced local expression of the vasculogenic and regenerative cytokines vascular endothelial cell growth factor (VEGF) and hypoxia-inducible factor 1-alpha (HIF-1α) has been reported in diabetic tissues following injury [4,5].

A variety of treatment approaches that seek to address the specific deficiencies present in diabetic wounds have been developed. Cell-based therapies, in particular, represent an appealing treatment paradigm, as they potentially contribute both cytokines and a cellular framework to the tissue regeneration process. In support of this approach, multiple cell-based products delivering fibroblasts or fibroblast-keratinocyte mixtures have a proven clinical efficacy for the treatment of diabetic wounds [6]. Moreover, advanced biomaterials are being developed to optimize cell survival and functionality within the harsh wound environment [7-9].

Adipose-derived mesenchymal stem cells (ASCs) are an especially appealing therapeutic cell source because of their multipotency, paracrine secretion of growth factors and cytokines [10], and demonstrated efficacy in models of wound healing and ischemic revascularization [11,12]. Additionally, adipose tissue presents a reservoir of readily available cells, which can be easily harvested and directly employed for autologous clinical application [13]. This methodology obviates the need for *ex vivo* cell expansion while avoiding the immunogenic concerns associated with allogeneic cell transplantation. The value of autologous cell therapies in patients with diabetes may be limited, however, as stem cell phenotypes can be negatively impacted by diabetes [14,15], and diabetic ASCs display an impaired neovascular potential *in vitro*[16]. Nonetheless, the therapeutic efficacy of diabetic ASCs within cutaneous wounds remains unclear [17].

Single-cell analytical approaches facilitate the in-depth exploration of cell heterogeneity and subpopulation dynamics, and we have recently used this technique to characterize stem cell phenotypes [18] and enhance ASC functionality via predictive subpopulation enrichment [19]. Importantly, determining the effect of diabetes on ASCs with single-cell granularity is the only way to differentiate between a global effect on cell functionality and a more selective subpopulation-level influence. This information is critically important for the design of effective autologous ASC-based therapies in patients with diabetes, as a global cell dysfunction would demand *ex vivo* modulation prior to application, whereas subpopulation-level effects may be overcome through more selective cell enrichment. In this study, we examine the impact of diabetes on the ASC niche as well as the ability of ASCs to promote neovascularization and wound healing when delivered within a biomimetic hydrogel scaffold developed in our laboratory [7,8]. Finally, we interrogate these cells on a single-cell level to characterize ASC population dynamics associated with this pathologic state.

## Methods

### Animals

Wild-type (WT) (C57BL/6) and type 2 diabetic (DM2) mice (BKS.Cg-*m* +/+Lepr^*db*^) were purchased from Jackson Laboratories (Bar Harbor, ME, USA) to study the effect of diabetes on ASC physiology. Selected assays were also performed on ASCs isolated from WT mice following induction of type 1 diabetes (DM1) via streptozotocin injection (STZ) (Sigma-Aldrich, St. Louis, MO, USA) as previously described [20]. Only mice with blood glucose levels of greater than 350 mg/dL were considered diabetic and used for further analysis. All protocols were approved by the Stanford Administrative Panel on Laboratory Animal Care.

### ASC niche analysis

WT and DM2 murine inguinal fat pads were harvested and manually disrupted for real-time quantitative polymerase chain reaction as described below.

### ASC harvest and culture

ASCs were isolated from WT, DM1, and DM2 murine inguinal fat pads, minced, and digested for 1 hour at 37°C using collagenase I (Roche Applied Science, Indianapolis, IN, USA). The reaction was stopped with the addition of supplemented media, pelleted via centrifugation, and subjected to erythrocyte lysis in accordance with the instructions of the manufacturer (Sigma-Aldrich). The remaining cells were pelleted again, forming the stromal vascular fraction (SVF). The SVF was either cultured under standard conditions (37°C in 5% CO_2_) in Dulbecco’s modified Eagle’s medium (DMEM) with 10% fetal bovine serum (FBS) and 1% penicillin/streptomycin (Life Technologies, Grand Island, NY, USA) containing 1 g/L of glucose to purify for ASCs or used immediately for flow cytometric/single-cell analyses. Cultured cells were used at or before passage 2, and all analyses were run in triplicate unless otherwise stated.

### *In vitro* Matrigel tubulization assays

PKH26-labeled WT and DM2 ASCs alone or mixed with calcein-labeled human umbilical vein endothelial cells (HUVECs) (Life Technologies) were cultured for 12 hours under hypoxic conditions on a 24-well plate (4 × 10^4^ cells per well) coated with growth factor-reduced Matrigel (BD Biosciences, Franklin Lakes, NJ, USA). ASC and HUVEC tubule counts were determined in five random high-power fields per well, respectively, by using an inverted Leica DMIL microscope (Leica Microsystems, Wetzlar, Germany).

### *In vivo* Matrigel plug assay and CD31 immunohistochemistry

WT or DM2 ASCs (8 × 10^5^) (cultured not more than two passages) were suspended in 250 μL of growth factor-reduced Matrigel (BD Biosciences) and injected in a subcutaneous fashion on the dorsum of WT mice (n = 4). Plugs were harvested at day 10, and 7-μm-thick frozen sections were immunohistochemically stained for the commonly used vascular marker platelet/endothelial cell adhesion molecule 1 (PECAM1/CD31, a transmembrane glycoprotein expressed on the surface of platelets, endothelial cells, and subsets of hematopoietic cells but particularly concentrated at the intercellular junctions of endothelial cells), followed by ImageJ (National Institutes of Health, Bethesda, MD, USA) quantification [7,21].

### *In vitro* adipogenic differentiation

WT and DM2 ASCs were seeded in standard six-well tissue culture plates (1.5 × 10^5^ cells per well), and adipogenic differentiation medium—consisting of DMEM (1 g/L glucose), 10% fetal bovine serum, 1% penicillin/streptomycin, 10 μg/mL insulin, 1 μM dexamethasone, 0.5 mM methylxanthine, and 200 μM indomethacin—was added after cell attachment. Oil red O staining was performed after 7 days of incubation.

### *In vitro* osteogenic differentiation

WT and DM2 ASCs were seeded in standard six-well tissue culture plates (1.0 × 10^5^ cells per well) and grown to at least 80% confluence before being cultured in osteogenic differentiation medium, which consisted of DMEM (1 g/L glucose) supplemented with 10% FBS, 1% penicillin/streptomycin, 100 μg/mL ascorbic acid, and 10 mM β-glycerophosphate. Photometric quantification of Alizarin red stain was performed after 14 days to assay extracellular mineralization as previously described [22].

### *In vitro* hydrogel bioscaffold seeding

WT and DM2 ASCs (1 × 10^5^) were suspended in 15 μL of growth media and seeded within a previously described 5% collagen-pullulan hydrogel bioscaffold [7,8]. Seeded scaffolds were placed in growth media and incubated at 37°C in 5% CO_2_ prior to proliferation and survival analyses and RNA/protein harvest.

### *In vitro* proliferation and survival

After hydrogel bioscaffold seeding, a live-dead assay (Live/Dead Cell Viability Assay) was performed at multiple time points to assess WT and DM2 ASC viability in accordance with the instructions of the manufacturer (Life Technologies). ASC proliferation was compared between hydrogel-seeded WT and DM2 cells at multiple time points by using an MTT assay (Vybrant MTT Cell Proliferation Assay Kit; Invitrogen, Grand Island, NY, USA).

### Real-time quantitative polymerase chain reaction

Total RNA was isolated from ground WT and DM2 fat pads or hydrogel-seeded ASCs by using an RNeasy Mini Kit (Qiagen, Germantown, MD, USA) and transcribed to cDNA (Superscript First-Strand Synthesis Kit; Invitrogen). Real-time quantitative polymerase chain reactions (qPCRs) were performed by using TaqMan gene expression assays (Applied Biosystems, Foster City, CA, USA) for murine *Mmp-9* (matrix metalloproteinase 9, Mm00442991_m1), *Cxcl-12* (stromal cell-derived factor-1/Sdf-1, Mm00445552_m1), *Vegf-a* (vascular endothelial growth factor-A, Mm01281447_m1), *Eng* (endoglin, Mm00468256_m1), *Hgf* (hepatocyte growth factor, Mm01135193_m1), *Mmp-3* (matrix metalloproteinase 3, Mm00440295_m1), *Cxcr-4* (chemokine receptor 4, Mm01292123_m1), *Fgf-2* (fibroblast growth factor 2, Mm00433287_m1), *Fgfr-2* (fibroblast growth factor receptor 2, Mm01269930_m1), *Pdgf-a* (platelet-derived growth factor-A, Mm01205760_m1), *Pdfgr-a* (platelet-derived growth factor receptor-A, Mm01205760_m1), and *Angpt-1* (angiopoietin 1, Mm00456503_m1) by using a Prism 7900HT Sequence Detection System (Applied Biosystems). Expression levels of the target genes were normalized to *Actb* (beta actin, Mm01205647_g1) or *B2m* (beta-2-microglobulin, Mm00437764_m1).

### Angiogenesis array

Angiogenic cytokine protein production from hydrogel-seeded WT and DM2 ASCs was quantified by using a Mouse Angiogenesis Array Kit (R&D Systems, Minneapolis, MN, USA). Pixel density of each spot in the array was quantified and normalized to controls by using ImageJ (National Institutes of Health).

### *In vivo* murine ischemia model

A model of graded soft tissue ischemia was created on the dorsum of WT mice, as described previously [23]. Briefly, a full-thickness three-sided peninsular flap was elevated, and a thin silicone sheet was inserted to separate the skin from the underlying fascia. A size-matched hydrogel bioscaffold was inlaid between the silicon sheet and skin flap either alone or following the seeding of 2.5 × 10^6^ WT or diabetic (type 2 or 1) ASCs (cultured not more than two passages) (n = 5). Unseeded scaffolds were used as controls. The skin flap was then sutured into place, and the demarcating necrotic area was quantified 10 days post-op. The proximal surviving flap was processed for neovascular growth via CD31 immunohistochemistry.

### Microfluidic single-cell gene expression analysis

ASCs isolated from freshly harvested WT, DM2, and DM1 SVF (obtained as described above) by using the surface marker profile CD45^-^/CD31^-^/CD34^+^ (to exclude contaminating CD45^+^ hematopoietic and CD31^+^ endothelial cells found within the SVF and to select for a putatively stem-like subset of CD34^+^ cells [22,24]) were analyzed and sorted as single cells by using a Becton Dickinson FACSAria flow cytometer (Becton Dickinson, Franklin Lakes, NJ, USA) into 6 μL of lysis buffer. Propodium iodide exclusion was used to ensure that only live cells were sorted. Reverse transcription and low cycle pre-amplification were performed by using Cells Direct (Invitrogen) with TaqMan assay primer sets (Applied Biosystems) in accordance with the specifications of the manufacturers. cDNA was loaded onto 96.96 Dynamic Arrays (Fluidigm, South San Francisco, CA, USA) for qPCR amplification by using Universal PCR Master Mix (Applied Biosystems) with a uniquely compiled TaqMan assay primer set as previously described [18].

### Statistical analysis

Results are presented as mean ± standard error of the mean. Data analysis was performed by using a Student *t* test. Results were considered significant for *P* values of not more than 0.05. For single-cell transcriptional data, a Kolmogorov-Smirnov (K-S) test was used to compare empirical distributions, followed by an adaptive fuzzy c-means clustering algorithm as previously described [18].

## Results

### Diabetes negatively impacts the ASC niche and impairs ASC promotion of neovascularization

**Figure 1:**
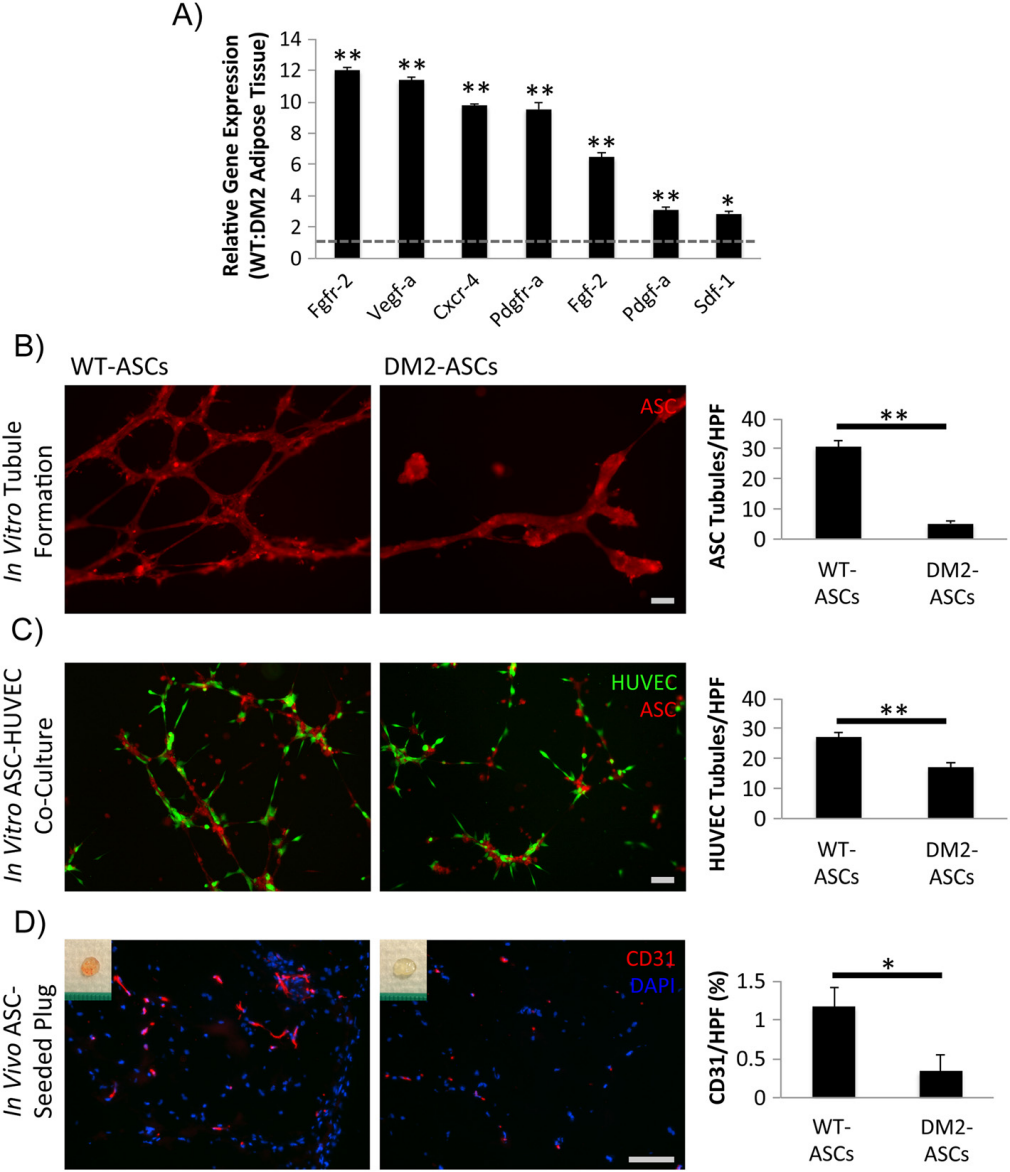
**Adipose-derived mesenchymal stem cell (ASC) niche characterization and i*n vitro *and *in vivo *analyses of ASC neovascular potential. (A)** Transcriptional profile of type 2 diabetes mellitus (DM2) and wild-type (WT) murine fat pads. A dysfunctional *in situ* signaling environment was observed in the setting of diabetes. **(B)** Matrigel culture of WT and DM2 ASCs under hypoxic conditions. Tubules per high-power field (HPF) were quantified as a surrogate for direct ASC vasculogenic potential. **(C)** Matrigel co-culture of ASCs and human umbilical vein endothelial cells (HUVECs) under hypoxic conditions. HUVEC tubules per HPF were quantified as a measure of ASC-mediated endothelial network formation. **(D)** CD31 staining to quantify *in vivo* Matrigel plug vascularity following seeding with either WT or DM2 ASCs. Insets provide gross images of explanted plugs. Scale bar = 50 μm. *P ≤0.05, **P <0.01.

To determine the impact of diabetes on the ASC niche *in situ*, the transcriptional profiles of DM2 and WT murine fat pads were analyzed. Indicative of a dysfunctional signaling environment *in situ*, the expression of multiple growth factors and cytokines (*Vegf-a*, *Fgf-2*, *Pdfg-a*, and *Sdf-1*; *P* <0.02) as well their associated receptors (*Cxcr-4*, *Fgfr-2*, and *Pdgfr-a*; *P* <0.01) was significantly decreased in the setting of diabetes (Figure 1A).

To next assess the potential of isolated diabetic ASCs to support the generation of vascular structures, we conducted a Matrigel tubule formation assay. When seeded alone, DM2 ASCs displayed significantly fewer tubular structures compared with WT cells (5.2 versus 30.6 tubules per HPF, respectively; *P* <0.001) (Figure 1B), suggesting a deficient response to extracellular matrix stimuli. ASCs are known to mediate angiogenesis mainly through paracrine mechanisms, however, as opposed to direct endothelial differentiation [25]. To analyze the ability of ASCs to promote endothelial sprouting, WT and DM2 ASCs were co-seeded with HUVEC cells by using the same model. Interestingly, although both ASC groups similarly localized around the HUVECs, consistent with their perivascular nature, the DM2 ASCs supported significantly less HUVEC tubule formation compared with WT ASCs (16.9 versus 27.1 tubules per HPF; *P* = 0.001) (Figure 1C), indicating that diabetic ASCs possess a reduced stimulatory capacity.

**Figure 2:**
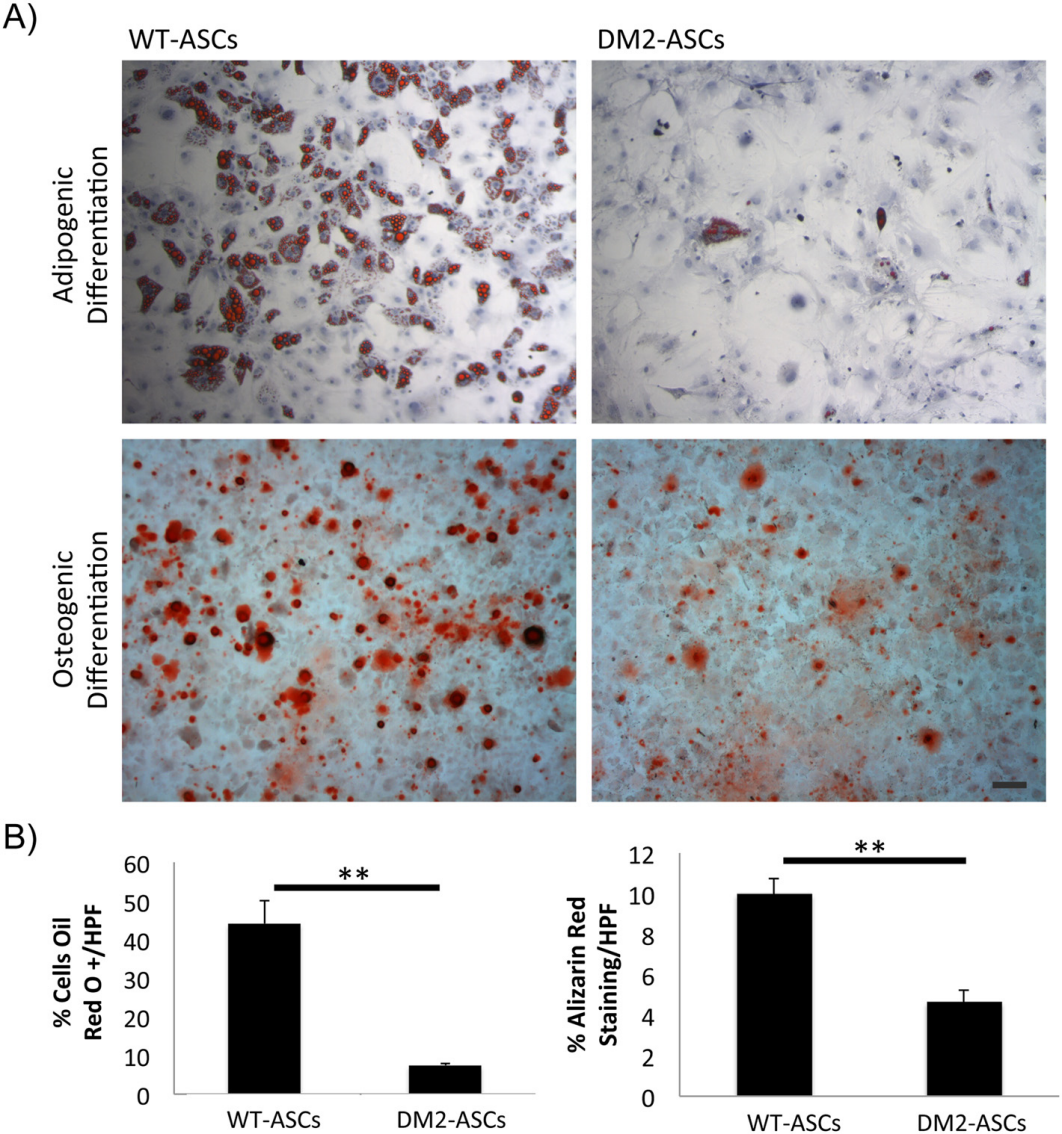
**Adipose-derived mesenchymal stem cell (ASC) adipogenic and osteogenic differentiation. (A)** Representative images and **(B)** quantification of Oil red O and Alizarin red staining following adipogenic and osteogenic differentiation of WT and DM2 ASCs. Scale bar = 50 μm. **P <0.01.

To determine whether the observed *in vitro* findings translated into impaired *in vivo* neovascularization, we performed an assay in which Matrigel plugs seeded with DM2 or WT ASCs were implanted into subcutaneous pockets on the dorsum of WT mice, and new vessel formation was histologically assessed after 10 days (a time point at which neovascular processes are active following injury [26]). Consistent with our *in vitro* findings, plugs containing DM2 ASCs displayed significantly reduced levels of vascularization (as determined by CD31 immunohistochemistry) compared with plugs seeded with WT ASCs (0.38 versus 1.16% CD31 staining per HPF; *P* = 0.02) (Figure 1D). Further exploring the effect of diabetes on ASC stemness, DM2 ASCs also displayed impairments in adipogenic and osteogenic differentiation *in vitro* (Figure 2), consistent with a loss of cell stemness and a phenotypic imprinting despite correction of the glucotic environment. Together, these data demonstrate that diabetes significantly impairs the stemness and potential of ASCs to promote neovascularization both *in vitro* and *in vivo*.

### Optimization of *ex vivo* environment does not restore diabetic ASC function

**Figure 3:**
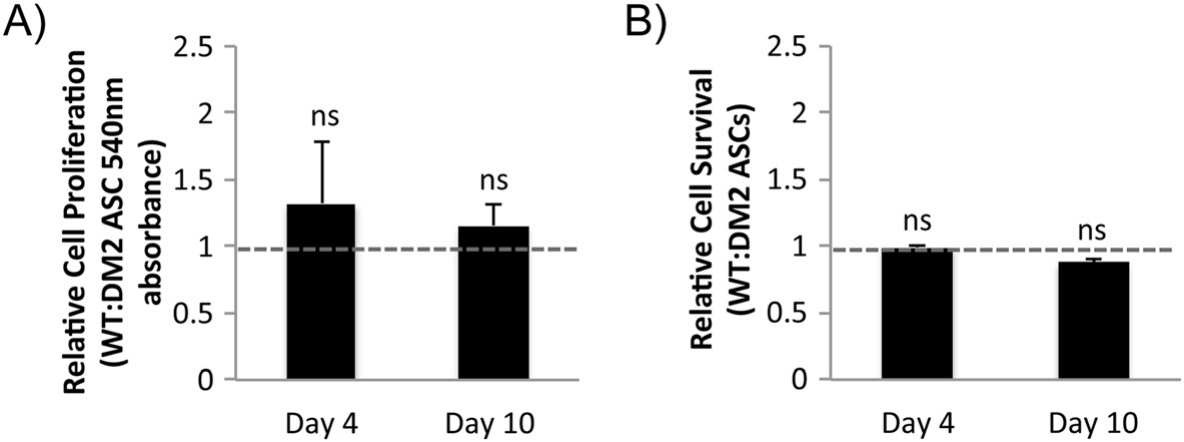
**ASC behavior following *in vitro *hydrogel seeding. (A,B)** Wild-type (WT) and diabetic (DM2) ASCs display similar rates of **(A)** proliferation and **(B)** survival *in vitro* following seeding within a three-dimensional bioscaffold. ns, not significant (*P* >0.05).

**Figure 4:**
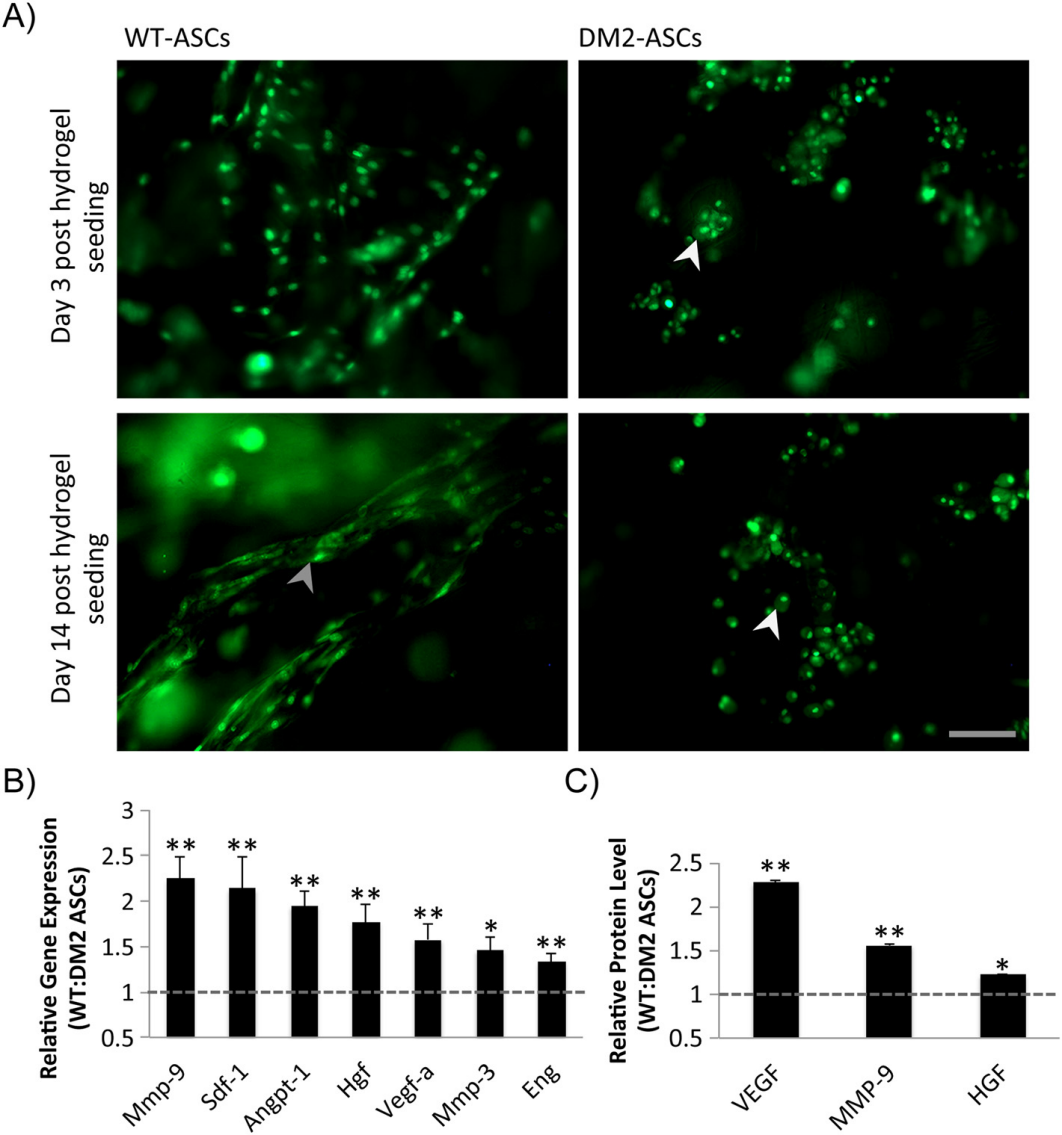
**Adipose-derived mesenchymal stem cell (ASC) incorporation and secretory function following hydrogel bioscaffold seeding. (A)** Wild-type (WT) and type 2 diabetes mellitus (DM2) ASCs at day 3 and 14 after hydrogel seeding. WT cells showed a uniform incorporation within the hydrogel and developed cell elongations and cytoplasmic extensions indicative of interactions with the bioscaffold (gray arrowhead). DM2 ASCs displayed a clumped distribution with rounded cell morphology (white arrowheads). **(B)** Reverse transcription-polymerase chain reaction and protein array **(C)** quantifying the relative expression of selected genes associated with vasculogenenesis and tissue remodeling in hydrogel-seeded WT versus DM2 ASCs. Scale bar = 50 μm. *P ≤ 0.05, **P <0.01. Angpt-1, angiopoietin 1; Eng, endoglin; Hgf, hepatocyte growth factor; Mmp-3, matrix metalloproteinase 3; Mmp-9, matrix metalloproteinase 9; Sdf-1, Stromal Cell-Derived Factor 1; Vegf, vascular endothelial cell growth factor.

The delivery of cells to a wound site is particularly challenging because of a hostile environment characterized by inflammation and poor nutritional support. We have recently developed a biomimetic hydrogel scaffold, which has been shown to support MSC survival, promote ‘stem-like’ features, and improve pro-angiogenic functionality [7,8]. To further characterize the effect of diabetes on the angiogenic potential of ASCs and to determine whether this function can be restored, we examined the cellular response to seeding in this biomimetic hydrogel. Although WT and DM2 ASCs displayed a similar seeding efficiency and proliferation capacity within the hydrogel (Figure 3), diabetic ASCs possessed a markedly abnormal morphology (Figure 4A). Specifically, DM2 ASCs appeared to be rounded and clumped and failed to form the cytoplasmic extensions seen with WT cells, indicative of a lack of cell interaction with the scaffold microenvironment. Importantly, this phenotypic dysfunction in DM2 ASCs was accompanied by a decrease in transcriptional expression and production of several vasculogenesis-related and tissue remodeling mediators, including VEGF, MMP-9, and HGF (*P* ≤0.03) (Figure 4B,C).

### Diabetic ASCs fail to improve ischemic wound healing *in vivo*

**Figure 5:**
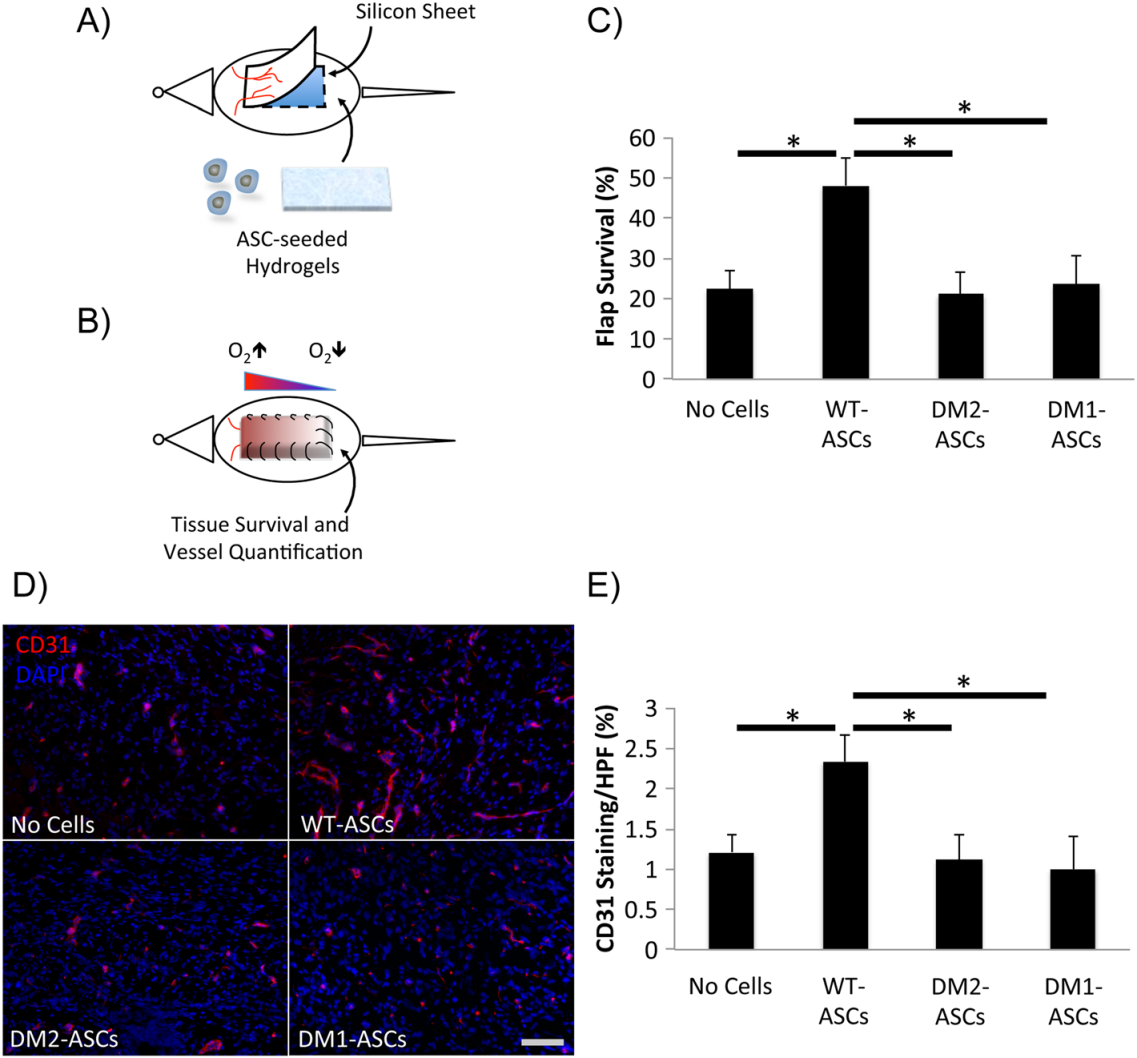
**Hydrogel delivery of ASCs to promote ischemic tissue flap survival. (A)** WT and DM (1 and 2) ASC-seeded hydrogels and unseeded controls were inset following creation of a full-thickness peninsular skin flap, **(B)** resulting in the creation of a reproducible ischemic gradient. **(C)** Tissue survival and vascularization as determined by **(D)** CD31 immunohistochemistry staining and **(E)** quantification were assessed at day 10. Scale bar = 50 μm. *P ≤0.05.

Diminished perfusion and neovascularization are important factors contributing to impaired diabetic healing; so to evaluate the regenerative potential of diabetic ASCs under representative conditions, cell-seeded biomimetic hydrogels were used in an adapted murine model of skin flap ischemia (Figure 5A,B) [27]. Although the application of WT ASCs showed a significant improvement in tissue survival at day 10 as compared with controls (48.0% versus 22.3% flap survival; *P* = 0.02), flaps treated with DM2 ASCs developed ischemic tissue necrosis at a level similar to that of the cell-free controls (21.0% versus 22.3% flap survival; *P* = 0.87) (Figure 5C). This difference in tissue survival correlated with a deficiency of DM2 ASCs in promoting new blood vessel formation within the surviving proximal flap as compared to WT ASCs (1.12% versus 2.34% CD31 staining per HPF; *P* = 0.04) (Figure 5D,E). This time point is of particular relevance as it is within the window of physiologic neovascularization that occurs following injury but prior to vascular regression that occurs as part of the normal tissue remodeling process [26]. Moreover, these results were recapitulated with ASCs isolated from streptozotocin-induced (DM1) mice, indicating that the observed findings were not model-specific, but rather reflect the impact of prolonged hyperglycemia on ASC physiology (Figure 5C-E). In aggregate, these data confirm the impaired vasculogenic potential of diabetic ASCs *in vivo*, despite delivery within a biomimetic scaffold.

### Diabetes selectively depletes a subpopulation of ASCs with a highly vasculogenic transcriptional profile

**Figure 6:**
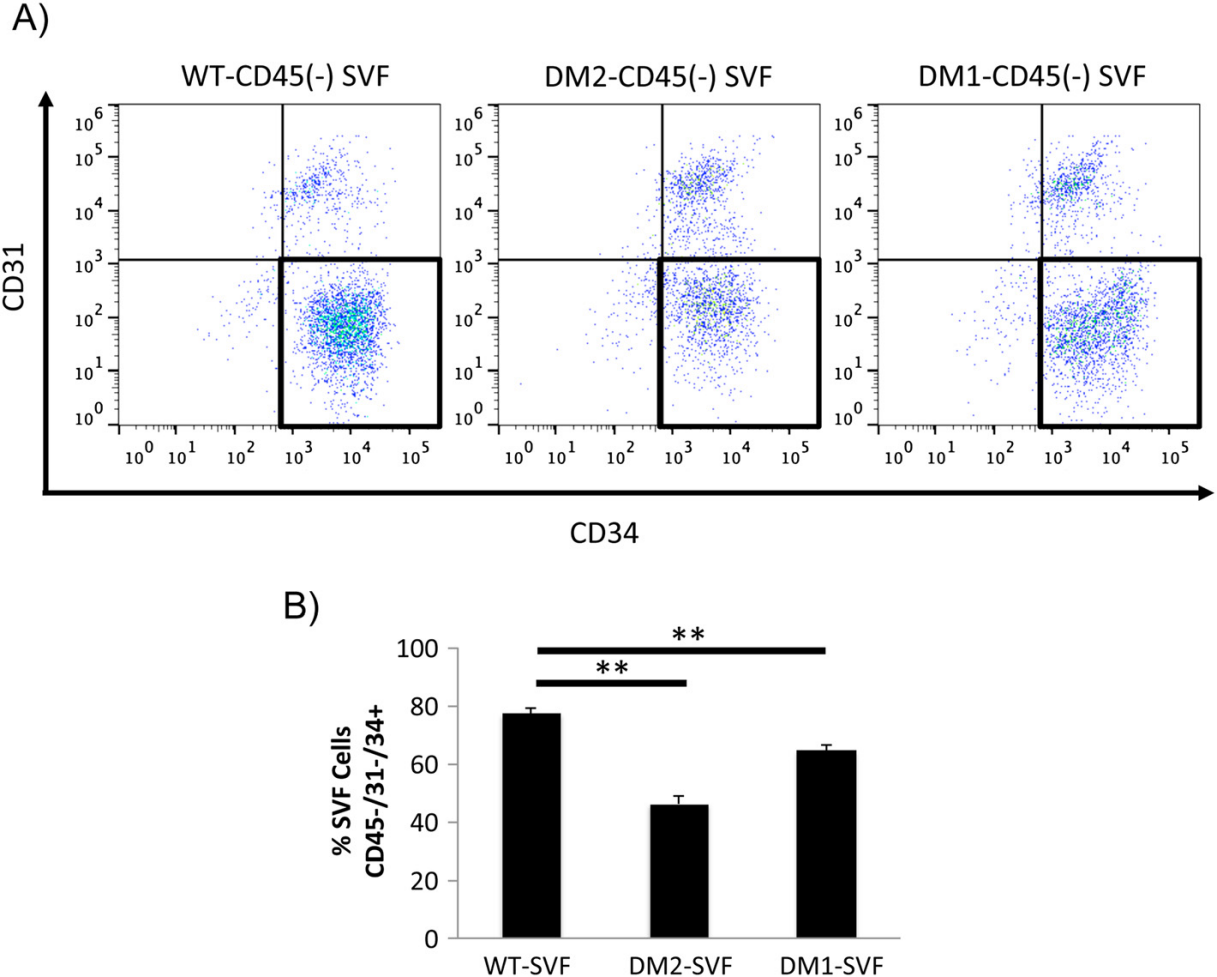
**Diabetic and wild-type (WT) stromal vascular fraction (SVF) cell surface marker analysis. (A)** Flow cytometric analysis determining the percentage of putative adipose-derived mesenchymal stem cells (ASCs) (CD45^-^/31^-^/34^+^ cells) within SVF obtained from diabetic (DM2 and DM1) and WT mice (CD45 and live/dead gating not shown). **(B)** Quantification of CD45^**-**^/31^**-**^/34^+^ ASCs in WT, DM2, and DM1 SVF reveals a significant depletion of ASCs in diabetic samples. **P ≤0.01.

Because cell culture and/or surface marker based enrichment of ASCs from the heterogeneous SVF is often employed prior to application, understanding the effect of diabetes on ASC population dynamics in the SVF, as well as following purification, is critical. To first determine the influence of diabetes on global ASC levels within the SVF, a flow cytometric analysis was performed on WT, DM2, and DM1 samples for the presence of putative ASCs (CD45^**-**^/CD31^**-**^/CD34^+^ cells). Indicative of a detrimental effect of diabetes on the global ASC population, both the DM2 and DM1 SVF displayed a reduced frequency of putative ASCs when compared with WT samples (*P* <0.01) (Figure 6).

Although this global ASC depletion provides an explanation for the dysfunction of diabetic SVF, a finer degree of granularity is needed to analyze purified ASCs for phenotypic differences, especially as there is evidence that even sorted ASCs consist of several subpopulations with varying levels of functionality [19]. To determine the effect of diabetes on ASC subpopulation dynamics, a single-cell interrogation of WT, DM2, and DM1 ASCs was conducted. For this, we employed microfluidic-based single-cell gene expression analysis as previously described [18]. Transcriptional profiles were simultaneously evaluated for approximately 70 gene targets across 75 individual cells per group, specifically looking at genes relating to stemness, vasculogenesis, and tissue regeneration (Additional file 1: Table S1). These cells were primary ASCs isolated based on a primitive surface marker definition (CD45^-^/CD31^-^/CD34^+^) intended to exclude contaminating hematopoietic and endothelial cells found within the SVF.

**Figure 7:**
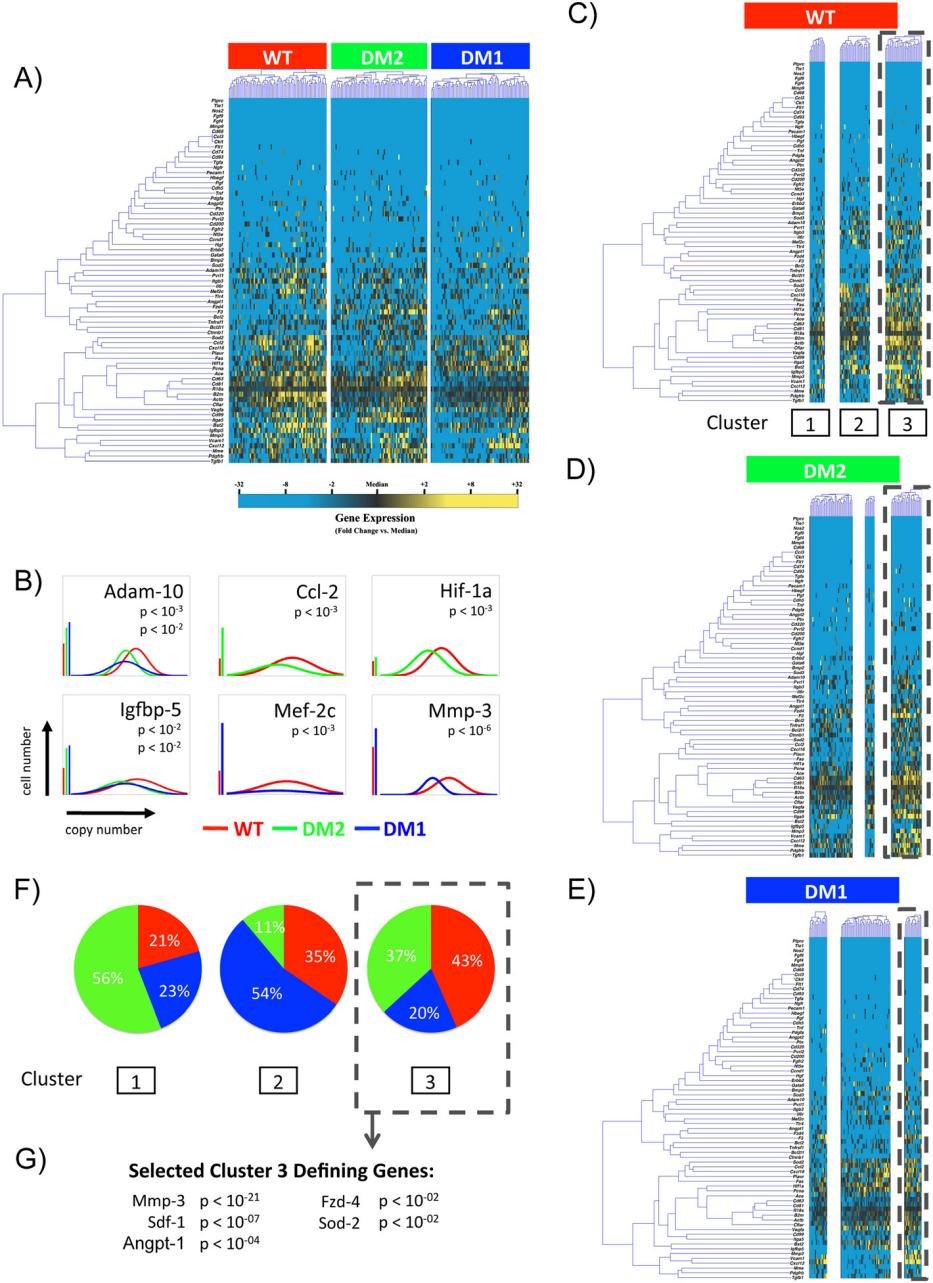
**Single-cell transcriptional analysis of CD45**^**-**^**/CD31**^**-**^**/CD34**^**+ **^**ASCs. (A)** Hierarchical clustering of cells from wild-type (WT; left), db/db diabetic (DM2; middle), and STZ-induced diabetic (DM1; right) mice. Gene expression is presented as fold change from median on a color scale from yellow (high expression, 32-fold above median) to blue (low expression, 32-fold below median). See Additional file 2: Figure S1 for complete dataset. **(B)** Differentially expressed genes between wild-type (WT) and diabetic (db/db [DM2] or STZ [DM1]) cells identified by using non-parametric two-sample Kolmogorov-Smirnov testing. Twenty-one genes exhibited significantly different (*P* <0.01 following Bonferroni correction for multiple comparisons) distributions of single-cell expression between populations; six are illustrated here by using median-centered Gaussian curve fits. The left bar for each panel represents the fraction of qPCR that failed to amplify in each group. Curves and *p* values for each gene are shown only for those diabetic groups significantly different from wild-type cells. **(C)** wild-type, **(D)** DM2, and **(E)** DM1 cells based on the expression patterns of all 71 genes (k = 3). **(F)** Pie charts representing the fraction of ASCs comprising each cluster (WT [red], DM2 [green], and DM1 [blue]). **(G)** List of selected cluster 3-defining genes.

When this approach was used, cells isolated from both WT and diabetic mice were found to display significant heterogeneity at the single-cell level (Figure 7A and Additional file 2: Figure S1). Moreover, Kolmogorov-Smirnov analysis of these single-cell data identified multiple genes that were differentially expressed in WT versus type 2 or 1 diabetic ASCs, including genes related to tissue remodeling, vasculogenesis, and cell differentiation, such as the metalloproteinases *Mmp-3* and *Adam-10*, the chemokine *Ccl-2*, and the transcription factors *Hif-1a* and *Mef-2c* (*P* <0.01) (Figure 7B) [28-32]. To determine whether these differences were the result of changes in specific subpopulations of diabetic or WT ASCs, partitional clustering was applied to the super-set of transcriptional profiles encompassing WT and diabetic cells [18]. This analysis identified three distinct transcriptionally defined ASC subpopulations, which exhibited strikingly different profiles across conditions (Figure 7C-E). Interestingly, a distribution analysis of the cells comprising the third cluster revealed that there were considerably fewer diabetic ASCs compared with WT cells in this subpopulation (Figure 7 F), with this cluster characterized in part by the elevated expression of genes associated with vasculogenesis, such as *Angpt-1*, *Sdf-1*, *Mmp-3*, and *Fzd-4* (Figure 7G) [29,33-35]. These data suggest that this subpopulation may be primed to support vasculogenesis, and its depletion in the diabetic state provides a potential mechanism for the impairment of diabetic ASC vasculogenic potential.

## Discussion

In this work, we provide a comprehensive analysis of the reduced therapeutic potential of diabetic ASCs for the promotion of tissue neovascularization and, for the first time, identify the selective depletion of a subpopulation of cells with a pro-vasculogenic phenotype in this setting. One of the main clinical advantages of ASCs is their ease of use in an autologous fashion. However, the dysfunctional signaling environment observed in diabetic fat pads suggests that prolonged hyperglycemia has multidimensional detrimental effects on ASC niche homoeostasis. Moreover, the continuous impairment of diabetic ASC functionality following isolation and culture within an optimized biomimetic hydrogel scaffold significantly limits the translational potential of autologously derived ASCs in this population.

Supporting the irreversibility of these diabetes-related impairments, the hydrogel scaffold used in this work was created specifically to recapitulate the stem cell niche and has been previously shown to enhance the pro-vasculogenic cytokine secretion (VEGF, MCP-1, FGF-1, MMPs, etc.) of bone marrow-derived mesenchymal stem cells (BM-MSCs), improve BM-MSC survival and engraftment within the high-oxidative-stress environment of ischemic murine skin wounds, and ultimately increase the angiogenic effect and wound-healing potential of BM-MSC-based therapies [7,8]. Similarly, when ASCs are seeded in this hydrogel, they display an upregulation of vasculogenesis-related genes as well as an increased *in vivo* wound-healing potential (unpublished data). Given the proven efficacy of this hydrogel bioscaffold, the negligible beneficial effect of traditionally isolated diabetic ASCs when seeded in these constructs, particularly in promoting *in vivo* neovascular processes following ischemic injury, prompted us to explore the possibility of differential cell enrichment as a potential means to restore functionality.

Our recent work in the emerging field of microfluidic-based single-cell gene expression analysis supports the use of transcriptional-based cell clustering not only for the characterization of cell phenotypes [18] but also as a tool for functionality-based population enrichment [19]. Further justifying this approach, although others have shown that diabetes can negatively impact the therapeutic potential of the heterogeneous SVF for wound-healing applications [36], it remained unclear from this work whether this deficiency was the result of a global depletion of ASCs within the adipose tissue of diabetic animals or alternatively the manifestation of a changing membrane phenotype associated with impaired cell functionality.

Casting a wide net in our surface marker-based definition of ASCs, we observed a depletion of putative ASCs (CD45^-^/CD31^-^/CD34^+^ cells) within the diabetic SVF, which was consistent with the signaling dysfunction seen in this environment. The presence of a global ASC depletion does not exclude further phenotypic dysfunction, however, and the microfluidic-based approach allowed us to analyze the transcriptional profile of ASCs for phenotypic heterogeneity independent of *a priori* classifications. Thus, the depletion of a putatively vasculogenic ASC subpopulation in diabetes identified here not only provides further insight into the impaired phenotype of these cells but also suggests that the regenerative effects of healthy ASCs may be dependent upon a critical subpopulation of cells. Based upon these findings, work is currently ongoing to confirm the therapeutic efficacy of the identified subpopulation as well as to determine whether prospective enrichment of the depleted diabetic ASC subpopulation restores cell functionality.

## Conclusions

The impaired therapeutic properties displayed by diabetic ASCs are likely related to the selective depletion of a highly vasculogenic subpopulation of cells. Although these data suggest that the utility of unaltered autologous ASCs for cell-based therapies in patients with diabetes is limited, differential cell isolation techniques designed to enrich for depleted subpopulations are areas of future study that may potentially improve diabetic ASC function.

## Abbreviations

Angpt-1: angiopoietin 1; ASC: adipose-derived mesenchymal stem cell; DM: diabetes mellitus; DM1: type 1 diabetes mellitus; DM2: type 2 diabetes mellitus; DMEM: Dulbecco’s modified Eagle’s medium; FBS: fetal bovine serum; Fgf-2: fibroblast growth factor 2; Fgfr-2: fibroblast growth factor receptor 2; Hgf: hepatocyte growth factor; Hif-1α: hypoxia-inducible factor 1-alpha; MMP: matrix metalloproteinase; Pdgf-a: platelet-derived growth factor-A; qPCR: quantitative polymerase chain reaction; SVF: stromal vascular fraction; Vegf: vascular endothelial cell growth factor; WT: wild-type.

## Competing interests

Funding for the stem cell research conducted in our laboratory has been provided by the Hagey Family Endowed Fund in Stem Cell Research and Regenerative Medicine, the Armed Forces Institute of Regenerative Medicine (US Department of Defense), the National Institutes of Health (R01-DK074095, R01-EB005718, and R01-AG025016), and the Oak Foundation. GCG is listed on the following patents assigned to Stanford University: (1) Intelligent Biodegradable Pullulan Regenerative Matrix for Tissue Engineering and (2) Efficient stem cell delivery into biomaterials using a novel capillary driven encapsulation technique. RCR and DD are also listed on patent (2). The other authors declare that they have no competing interests. Parts of this study were presented at the 7th Annual Academic Surgical Congress on 14-16 Feb. 2012.

## Authors’ contributions

RCR and MS contributed equally to the idea generation, experimental work, and manuscript preparation. MJ, DD, RK, MTC, JL, AR-D, SR, ZNM, MSH, JR, and MR contributed to the experimental work and manuscript preparation. GCG guided the idea generation, experimental work, and manuscript preparation. All authors contributed to the idea generation, design, and completion of this work and read and approved the final manuscript. 

## Supplementary Material

Additional file 1: Table S1 Gene names and assay IDs for microfluidic single-cell gene expression analysis. Genes specifically relating to stemness and vasculogenesis were chosen, in addition to selected control, cell cycle, and surface marker-related probes. Click here for file

Additional file 2: Figure S1 Whisker plots presenting raw quantitative polymerase chain reaction (qPCR) cycle threshold (Ct) values for each gene across all wild-type (WT) and diabetic adipose-derived mesenchymal stem cells. Individual dots represent single gene/cell qPCR reactions, and increased Ct values correspond to decreased mRNA content. Ct values of 40 were assigned to all reactions that failed to achieve detectable levels of amplification within 40 qPCR cycles. Cells isolated from WT, db/db (DM2), and STZ (DM1) diabetic mice are colored in red, green, and blue, respectively. DM1, type 1 diabetes mellitus; DM2, type 2 diabetes mellitus. Click here for file
