# Supplementary figures and images for: Diabetes impairs the angiogenic potential of adipose-derived stem cells by selectively depleting cellular subpopulations
Source: Stem Cell Res Ther. 2014 Jun 18;5(3):79. doi: 10.1186/scrt468 (PMC4097831; doi:10.1186/scrt468)

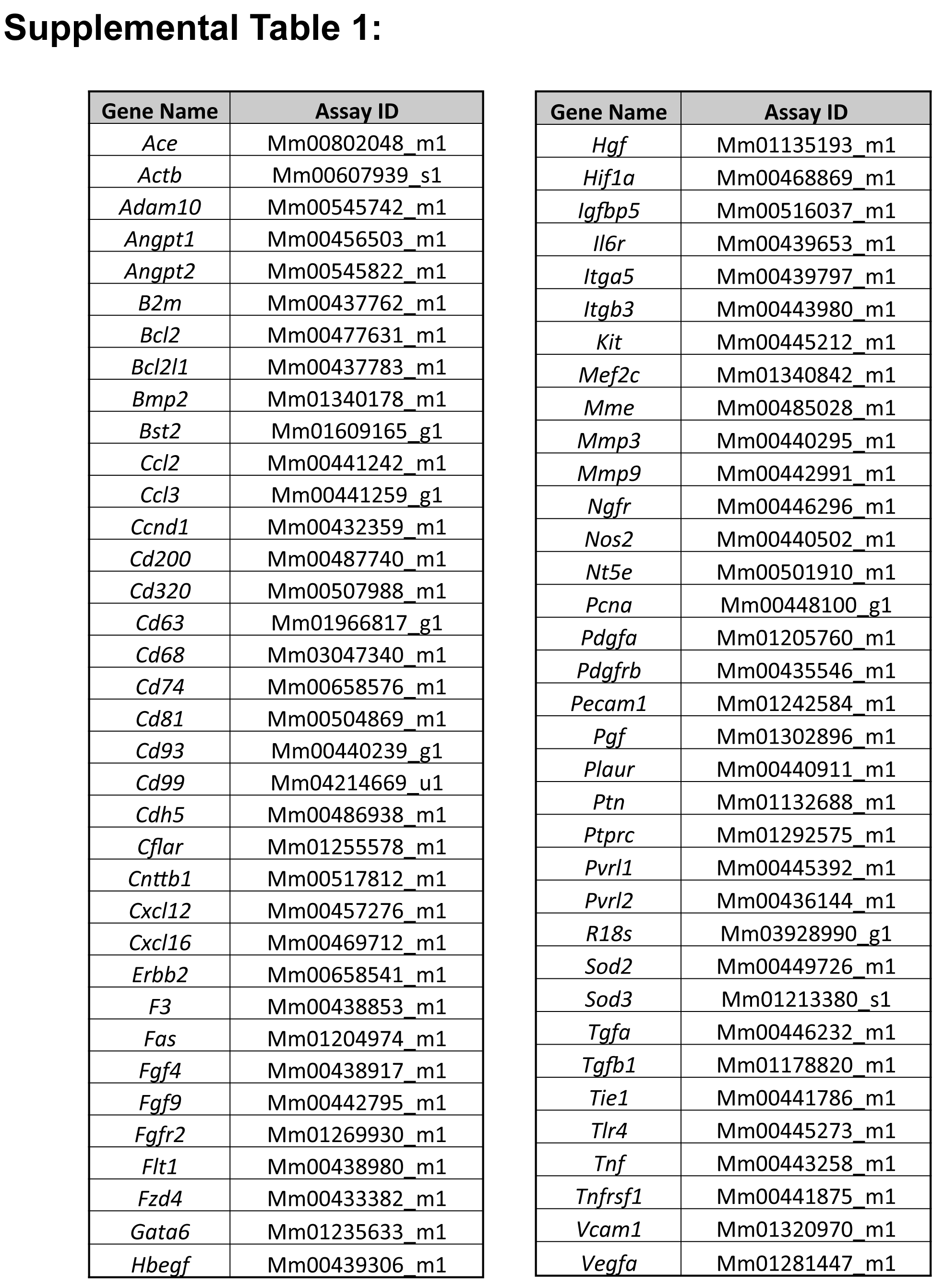

Supplement: Additional file 1: Table S1 — Gene names and assay IDs for microfluidic single-cell gene expression analysis. Genes specifically relating to stemness and vasculogenesis were chosen, in addition to selected control, cell cycle, and surface marker-related probes. [file scrt468-S1.tiff]

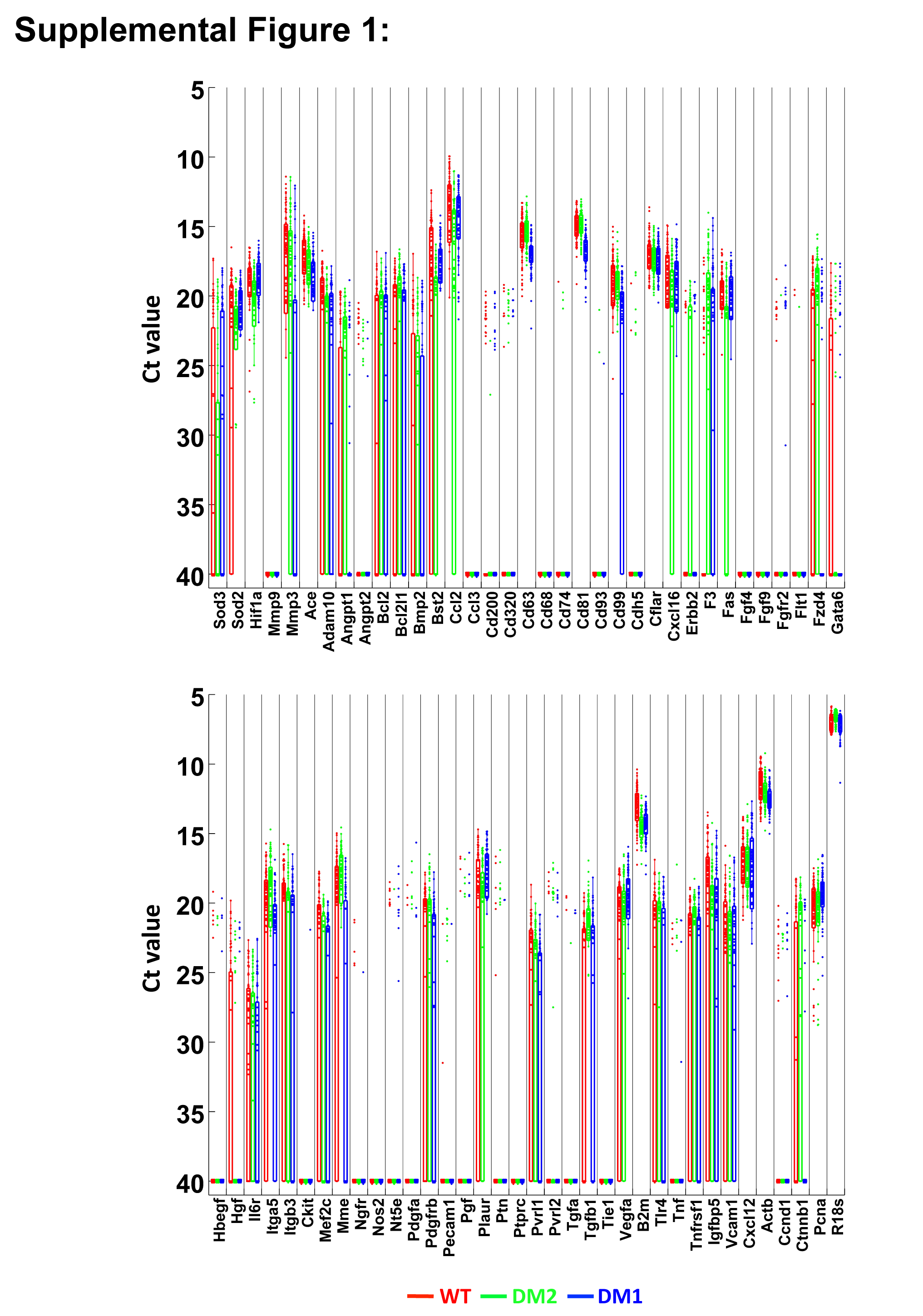

Supplement: Additional file 2: Figure S1 — Whisker plots presenting raw quantitative polymerase chain reaction (qPCR) cycle threshold (Ct) values for each gene across all wild-type (WT) and diabetic adipose-derived mesenchymal stem cells. Individual dots represent single gene/cell qPCR reactions, and increased Ct values correspond to decreased mRNA content. Ct values of 40 were assigned to all reactions that failed to achieve detectable levels of amplification within 40 qPCR cycles. Cells isolated from WT, db/db (DM2), and STZ (DM1) diabetic mice are colored in red, green, and blue, respectively. DM1, type 1 diabetes mellitus; DM2, type 2 diabetes mellitus. [file scrt468-S2.tiff]
